# Interesting Presentation of Oral Herpes Simplex Virus in a High-Risk Patient

**DOI:** 10.7759/cureus.16960

**Published:** 2021-08-06

**Authors:** Vixey Silva, Krystina Khalil, Steven Daveluy

**Affiliations:** 1 Dermatology, Michigan State University College of Osteopathic Medicine, East Lansing, USA; 2 Dermatology, St. Mary Mercy Livonia Hospital, Livonia, USA; 3 Dermatology, Wayne State University, Detroit, USA

**Keywords:** orolabial herpes, genital herpes, herpetic glossitis, herpes simplex virus, hsv-1, hsv-2

## Abstract

Orolabial and genital herpes are common conditions caused by herpes simplex virus (HSV) that affect many individuals. Although skin findings may mimic other conditions, an HSV infection may still be diagnosed clinically. We present a case of polymerase chain reaction (PCR) negative orolabial herpes in a young male with a high-risk social history. Although testing was negative, oral HSV was clinically diagnosed and the patient was adequately treated with IV acyclovir. Both clinical history and physical examination are essential to provide an accurate diagnosis even in cases presenting with a negative diagnostic test result.

## Introduction

Herpes simplex virus (HSV)-1 and HSV-2 are responsible for primary and recurrent orolabial and genital herpes, respectively. Although infrequent, HSV-2 also has the potential to cause orolabial herpes. Clinically, orolabial herpes presents as superficial ulcerative lesions of the tongue and surrounding oral mucosa [1]. Concurrently, painful perioral vesicles overlying an erythematous base may be present [1]. Up to 63% of people in the United States are HSV-1 seropositive and up to 60% are positive for HSV-2 antibodies [2]. The lesions visualized with HSV may mimic conditions such as Behçet’s syndrome, aphthous stomatitis, erythema multiforme, and Stevens-Johnson syndrome (SJS). Viral culture and polymerase chain reaction (PCR) may be performed to diagnose HSV if the presentation is not evident or a clinical outlier. However, a negative test result does not fully rule out HSV if suspicion remains high, making a careful history and clinical examination of utmost importance for diagnosis. We present a case of orolabial herpes predominantly on the tongue with a negative HSV PCR test result.

## Case presentation

**Figure 1:**
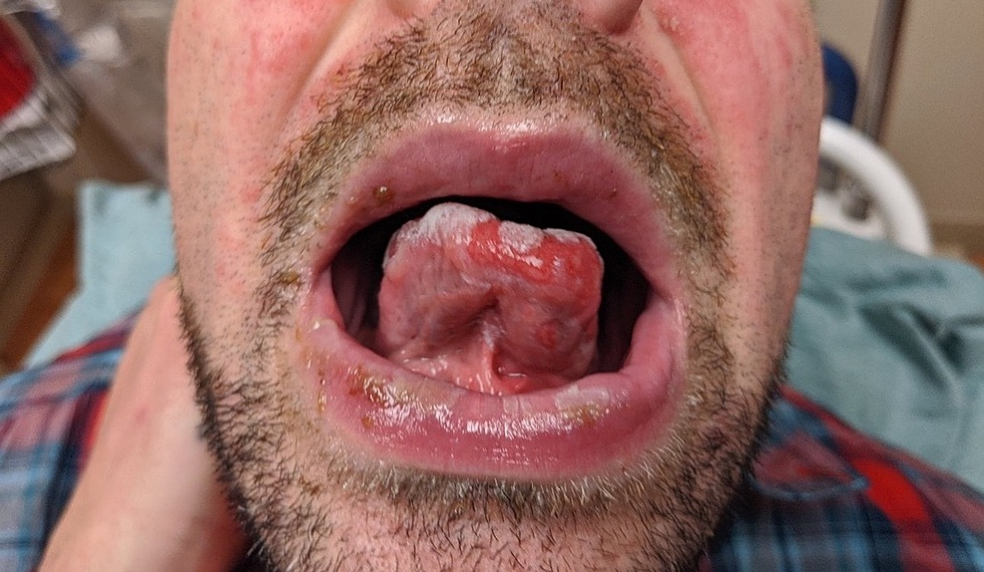
Lower lip exhibiting erosion with overlying slough and yellow crust. Hemorrhagic erosions of the tongue.

**Figure 2:**
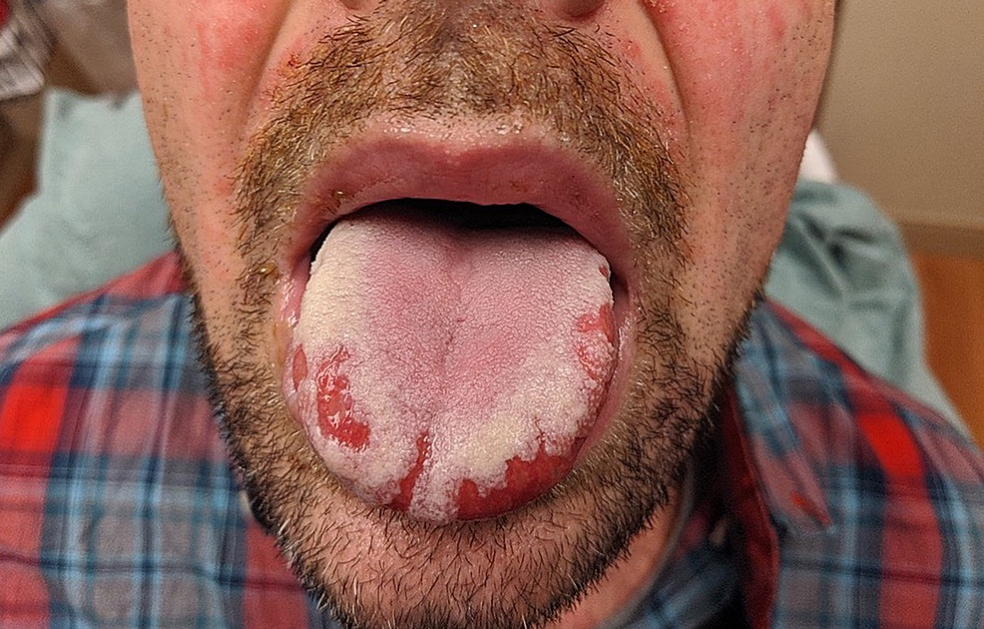
Erosions with scalloped borders of the tongue.

A 33-year-old male with a history of genital herpes and IV drug abuse presented with a painfully swollen tongue of two-day duration. He began oral acyclovir four days prior to presentation for recurrent genital herpes. Within two days of treatment, he experienced a “tingling” sensation of the tongue, leading to pain and edema. He had unprotected sexual intercourse and receptive oral sex with multiple male partners in the past month. Physical exam revealed large hemorrhagic erosions with scalloped borders along the lateral surface of the tongue. The large white lesion was not detachable upon scraping. The lower lip demonstrated erosions with overlying slough and yellow crust (Figures 1-2). Additionally, there were small punched-out ulcers with hemorrhagic crust on the base of the penile shaft. Lab workup included HIV, rapid plasma reagin (RPR), gonorrhea, chlamydia, hepatitis panel, and an oral HSV-1/HSV-2 PCR swab of the dorsal tongue; all were unremarkable. A clinical diagnosis of orolabial herpes was made. The patient was started on IV acyclovir 5 mg/kg every eight hours and began improving within 24 hours. He was discharged on day 2 of admission with oral acyclovir.

## Discussion

Following primary infection of HSV-1 or HSV-2, the virus lies dormant in the nervous system (e.g., trigeminal and sacral ganglia) causing recurrent breakouts, which can be frustrating for patients. Reactivation occurs in response to a trigger stimulus such as psychological stress, fatigue, sunlight, exposure to cold or heat, sexual intercourse, and immunosuppression [3]. Reactivation prodromal symptoms can include a sensation of burning or paresthesia at the site of inoculation prior to the appearance of vesicles. Diagnosis of orolabial herpes infection is typically based on clinical features, however, diagnosis can be confirmed with viral culture or PCR. Although PCR tests for HSV are both sensitive and specific, negative results do not rule out HSV infection, as evidenced by our case. 

During the initial work-up, our differential diagnosis included Behçet’s syndrome, aphthous stomatitis, erythema multiforme, and Stevens-Johnson syndrome (SJS) as they all present with oral and/or genital lesions. Aphthous ulcers do not involve the keratinized epithelium, such as the lower lip erosions seen in our patient, and Behçet’s syndrome is a multisystem inflammatory disease that also has ocular, neurological, and vascular manifestations. While oral ulcers are frequently the presenting sign of Behçet’s syndrome, our patient’s history of unprotected oral intercourse and rapid response to acyclovir is consistent with herpes infection [4]. Erythema multiforme can also be seen in association with HSV, however, it would present with targetoid skin lesions. Lastly, SJS can present with mucosal ulceration, but typically involves other areas of the skin with full-thickness necrosis as well as exposure to new medications [4].

Clinicians should always have a high index of suspicion for HSV in high-risk patients who present with similar signs and symptoms. Multiple tests may have to be completed just to yield accurate results [3]. Studies have reported an increased number of false-negative HSV PCR results during cerebrospinal fluid analysis in patients experiencing symptoms of infectious HSV. Two false-negative HSV PCR results for an elderly male presenting with altered mental status proved to be fatal. The autopsy was positive for HSV via immunohistochemistry despite negative PCR results [5]. In another case, a patient presenting with symptoms of HSV encephalitis required three HSV PCR tests before yielding a positive result. A potential explanation for false-negative test results are inhibitors such as heme factor, hemin, bilirubin, bile salts, lactoferrin, and immunoglobulins, as they interfere with PCR, leading to false-negative results [3]. Alternatively, falsely negative PCR results have been documented in patients with immunocompromised states in the setting of high viral loads [6]. Although HSV is self-limiting, acyclovir is one of the first-line treatments used for symptom resolution of acute flare-ups and chronic management [2].

## Conclusions

HSV is a prevalent virus with a relapsing and chronic course that affects a large portion of our society. This burdensome virus is known for its pathognomonic clinical presentation of numerous clear, fluid-filled vesicles upon an erythematous base. However, it is uncommon for HSV to primarily present on the tongue, straying from its common presentation on the lips. If testing is completed and a negative PCR result is obtained, this may complicate the picture, further leading clinicians down a path of misdiagnosis with delayed treatment. A careful history and physical examination are vital for early symptomatic management and to exclude clinical mimickers from the differential diagnosis.
